# Association of *ADIPOQ*, *OLR1* and *PPARGC1A* gene polymorphisms with growth and carcass traits in Nelore cattle

**DOI:** 10.1016/j.mgene.2015.02.001

**Published:** 2015-03-06

**Authors:** Patrícia D. da S. Fonseca, Fábio R.P. de Souza, Gregório M.F. de Camargo, Fernanda M.M. Gil, Diercles F. Cardoso, Larissa Zetouni, Camila U. Braz, Arione A. Boligon, Renata H. Branco, Lucia G. de Albuquerque, Maria E.Z. Mercadante, Humberto Tonhati

**Affiliations:** a Universidade Estadual Paulista (Unesp), Departamento de Zootecnia, Jaboticabal, SP 14884-900, Brazil; b Universidade Federal de Pelotas (UFPel), Departamento de Ecologia, Zoologia e Genética, Pelotas, RS 96010-900, Brazil; c Universidade Federal de Pelotas (UFPel), Departamento de Zootecnia, Pelotas, RS 96010-900, Brazil; d Instituto de Zootecnia, Centro APTA Bovinos de Corte, Sertãozinho, SP, Brazil

**Keywords:** *Bos taurus indicus*, Molecular markers, PCR-RFLP, SNP

## Abstract

In beef cattle farming, growth and carcass traits are important for genetic breeding programs. Molecular markers can be used to assist selection and increase genetic gain. The *ADIPOQ*, *OLR1* and *PPARGC1A* genes are involved in lipid synthesis and fat accumulation in adipose tissue. The objective of this study was to identify polymorphisms in these genes and to assess the association with growth and carcass traits in Nelore cattle. A total of 639 animals were genotyped by PCR-RFLP for rs208549452, rs109019599 and rs109163366 in *ADIPOQ*, *OLR1* and *PPARGC1A* gene, respectively. We analyzed the association of SNPs identified with birth weight, weaning weight, female yearling weight, female hip height, male yearling weight, male hip height, loin eye area, rump fat thickness, and backfat thickness. The *OLR1* marker was associated with rump fat thickness and weaning weight (P < 0.05) and the *PPARGC1* marker was associated with female yearling weight.

## Introduction

The beef cattle population, in tropical areas, is mainly composed of zebu breeds, particularly Nelore animals. The study of carcass quality traits is interesting for meat production and commercialization, because these traits may improve economically relevant factors such as carcass yield and amount of meat produced per hectare. The heritability of traits related to carcass quality, as loin eye area and fat thickness in carcass, has been shown to be high in Nelore cattle (Iti Yokoo et al., 2009, Pinheiro et al., 2011). However, carcass traits are measured late in life and the rate of genetic gain may be reduced. Therefore, the use of molecular markers for these traits has been proposed in an attempt to increase the accuracy of prediction and to reduce the generation interval (Ayres et al., 2010, Curi et al., 2005a, Curi et al., 2005b, Curi et al., 2006, Curi et al., 2009, Pablos de Souza et al., 2012, Souza et al., 2010).

The *ADIPOQ*, *OLR1* and *PPARGC1A* are some candidate genes that have been chosen to determine their influence on carcass traits because they are involved in lipid synthesis, metabolic synthesis, and fat accumulation in adipose tissue. The *ADIPOQ* gene (also known as adipocyte complement-related protein of 30 kDa) was first described (Scherer et al., 1995). Significant effects of markers in this gene have also been reported on carcass traits in pigs (Dai et al., 2006) and growth traits in goats (Fang et al., 2011). In cattle, the *ADIPOQ* gene is located on chromosome 1 in the vicinity of a QTL that affect marbling, loin eye area, fat thickness, intramuscular fat (Barendse, 2011, Morsci et al., 2006, Shin and Chung, 2013, Zhang et al., 2013, Zhang et al., 2014).

The *OLR1* gene is located in BTA5, it encodes the lectin-like oxidized low-density lipoprotein receptor, a protein that binds, internalizes and degrades oxidized low-density lipoproteins (oxLDL). Several studies with dairy cattle had shown significant effects of the marker rs109019599 (also descripted as g.8232C>A or C223A) in 3′ untranslated OLR1 gene with milk fat production (Ates et al., 2014, Khatib et al., 2006, Komisarek and Dorynek, 2009, Wang et al., 2013). According to Khatib et al. (2006), animals with the genotype AA have lower *OLR1* gene expression than other genotypes.

The *PPARGC1A* gene is located in BTA6 and is a co-activator of nuclear receptors and other transcription factors that regulate the adipogenesis (Samulin et al., 2008). Lee et al. (2012) showed that the *PPARGC1A* gene is related to a variety of porcine biological processes associated with the quality of muscle fiber formation. Significant associations between the *PPARGC1A* gene and milk production traits have been described in dairy cattle (Ates et al., 2014, Khatib et al., 2007, Weikard et al., 2005) and also carcass and growth traits in beef cattle (Li et al., 2014, Sevane et al., 2013, Shin and Chung, 2013).

The aim e of the present study was to verify the presence of the SNPs rs208549452, rs109019599 and rs109163366 in *ADIPOQ*, *OLR1* and *PPARGC1A* genes, respectively, in *Bos taurus indicus*, analyze the allelic and genotypic frequencies in three Nelore lines selected for growth and associate them with growth and carcass traits.

## Methods

### Animals

A total of 639 Nelore cattles derived from three selection lines, born and raised at Centro APTA Bovinos de Corte, Instituto de Zootecnia, Sertãozinho, São Paulo, Brazil, were used in this study. The animals are part of the Zebu and Caracu Selection Program which started in 1978, and is a didactic example of within-herd selection. In the selection and traditional lines (NeS and NeT), the animals are selected for higher yearling weight, and in the control line (NeC) the animals are selected for average yearling weight representing the animals' weight at the beginning of the selection program. The selection criteria used in this program were the male yearling body weight at 378 days of age and female yearling body weight at 550 days of age. The NeC and NeS were closed lines since their establishment. However, the NeT line eventually receives sires from other herds, including commercial ones.

Animals born between 2003 and 2009 were used in this study. Records from 104 animals (21 males and 83 females) from NeC, 189 animals (30 males and 159 females) from NeS and 346 animals (105 males and 241 females) from NeT were used.

The following traits were analyzed: birth weight (BW), weaning weight adjusted to 210 days of age (W210), females yearling weight adjusted to 550 days of age (W550), female yearling hip height (H550), males yearling weight adjusted to 378 days of age (W378), male yearling hip height (H378), loin eye area (LEA), backfat thickness (BF) and rump fat thickness (RF). The carcass traits were measured when both sexes were between 500 and 600 days of age. The LEA and BF were measured on transverse images in the longissimus muscle between the 12th and 13th rib and were used as an indicator trait of muscle tissue yield and finishing carcass. The RF was measured at the intersection between the gluteus medius and biceps femoris muscles located between the hook and pin bones. Real-time ultrasound images were acquired using two devices depending on the occasion: Aloka 500 V (Corometrics Medical Systems, Inc., Wallingford, CT) equipped with a 17.2-cm linear probe and 3.5-MHz transducer (Aloka Co. Ltd., Tokyo, Japan), and Pie Medical 401.347-Aquila (Esaote Europe BV, Maastricht, The Netherlands) equipped with an 18-cm linear probe and 3.5-MHz transducer. The images were stored and later interpreted using the Image Echo Viewer 1.0 (Esaote Europe BV). The results were rounded to one decimal.

### Genotyping and sequencing

Blood (5 mL) was collected from each animal by puncturing the jugular vein into Vacutainer tubes containing 7.5 mg EDTA and stored under refrigeration until the time of DNA extraction. Genomic DNA was extracted using the protocol of Zadworny and Kuhnlein (1990).

The sequence of the primers used for amplification of the fragments of *ADIPOQ*, *OLR1* and *PPARGC1A* genes, as well as the specific regions amplified and the annealing temperatures were described in the literature (Khatib et al., 2006, Khatib et al., 2007). The amplification conditions were denaturation at 95 °C for 5 min, followed by 30 cycles of denaturation at 95 °C for 1 min, annealing in temperature specific of each pair of primers for 45 s and extension at 72 °C for 1 min, and a final extension step at 72 °C for 5 min. For PCR, 100 ng genomic DNA, 1 μL of each primer (15 pmol), and the GoTaq Green Master Mix (Promega) were mixed in a final volume of 15 μL. The amplification reactions were carried out in a Gradient C-1000 thermocycler (BioRad).

To assess the existence of the SNPs rs208549452 in *ADIPOQ* gene, rs109019599 in *OLR1 gene* and rs109163366 in *PPARGC1A* gene in the Nelore population used in this study were made RFLP assays with the enzymes *Bsr*I, *Pst*I and *Nhe*I respectively. The digestion of each fragment was performed with 3 μL of the PCR product, 1 × reaction buffer, 1 × BSA, 1 U of the enzyme, and Milli-Q water mixed in a final volume of 10 μL. The mixture was incubated for 2 h at 65 °C for restriction with the enzyme *Bsr*I and at 37 °C to restriction with both *Pst*I and *Nhe*I enzymes, attending the manufacturer's specification for each enzyme (New England Biolabs, Inc., Ipswich, MA, USA). The digestion products of the *ADIPOQ* and *PPARGC1A* genes were separated on 2% agarose gel in 1 × TBE buffer (89 mM Tris–HCl, 2.5 mM EDTA and 89 mM boric acid, pH 8.3) at 90 V for 1 h and 40 min. The gels were visualized under a Gel–Doc transilluminator (BioRad), analyzed using the Kodak Image Analysis software, and stored for genotyping. For the *OLR1* gene, the digestion products were separated by electrophoresis on 8% polyacrylamide gel (8 mL acrylamide, 4 mL 10 × TBE, 40 μL Temed, 30 mL water, and 0.07 g ammonium persulfate). The polyacrylamide gels were run for 3 h and 48 min, and stained with silver nitrate.

Two samples of each identified genotype were sequenced by using both primers and a dideoxynucleotide (ddNTP) chain termination technique using the ABI PRISM BigDye Terminator Cycle Sequencing Ready Reaction kit (Applied Biosystems) in an automated ABI 3730 XL sequencer (Applied Biosystems). The sequences obtained were analyzed and visualized using the CodonCode Aligner available at (http://www.codoncode.com/aligner/download.htm).

### Statistical analysis

Allelic frequencies were compared between the three selection lines by Fisher's test using the Genepop 3.4 program (http://genepop.curtin.edu.au/). A P value ≤ 0.05 was considered to be significant.

The single marker regression with each trait was examined for genotyped animals using a mixed model analysis of variance with SAS (2008) software. The mixed model is described below:

$$y_{i}=X\beta +Z\mu +S_{j}a_{j}+e_{i}\text{.}$$

Where *yi* represents the phenotypic measurement for the *i*th animal, X is the incidence matrix relating fixed effects in *β* with observations in *y*, *Z* is the incidence matrix relating to random additive polygenic effects of animal in *μ* with observations in *y* and *S_j_* is the observed animal genotype for the jth SNP (coded as 0, 1 or 2 to represent the number of copies of the B allele), *a_j_* is the estimated SNP effect, and lastly *e_i_* is the random residual effect.

The model included the molecular markers (rs208549452, rs109019599, and rs109163366), contemporary group (year of birth × line, 1, …, 17), sex, and month of birth (September, October, November, December) as classificatory effects, age of dam and age at recording as linear covariates.

The percentage of the genetic variance accounted by the jth SNP was estimated according to the formula $\%Vj=100.\frac{2p_{j}q_{j}a_{j}^{2}}{\sigma_{g}^{2}}$ where p and q are the allele frequencies for the jth SNP estimated across the entire population, a_j_ is the estimated additive effect of the jth SNP on the trait under analysis, and *σ*_*g*_^2^ is the REML estimate of the (poly-) genetic variance for the trait.

## Results and discussion

Analysis of the mean weights and measures of the animals in each selection line showed lower mean growth traits for NeC animals when compared to the NeS and NeT lines. Means of H550 and H378 were greater in the NeS line, whereas BW, W210, W550, W378, rump fat thickness, backfat thickness and LEA means were higher in the NeT line (Table 1).

The genotypes observed in the agarose gel to marker rs208549452 in *ADIPOQ* gene after PCR-RFLP assays were TT (455 and 184 bp), CC (351, 184, and 104 bp), and TC (455, 351, 184, and 104 bp). To the marker rs109019599 in the *OLR1* gene the genotypes were AA (254 bp), CC (269 bp), and AC (269 and 254 bp) so that is not possible to visualize the fragment with 15 bp in the polyacrylamide gel. The marker rs109163366 in *PPARGC1A* gene identified the genotypes AA (358 bp), CC (324 bp), and AC (358 and 324 bp). The lengths of each fragment as such as the nucleotide substitution were confirmed by sequencing. The three sequences were deposited in GenBank under the accession numbers JQ775868, JQ775870, and JQ775869, respectively.

The frequencies obtained in the three selection lines are presented in Table 2. For the rs208549452 in *ADIPOQ* gene, the frequency of allele T was higher in the NeC and NeS lines, whereas C allele was higher in the NeT line. For the marker rs109019599 in *OLR1* gene, the frequency of the C allele was higher in the three selection lines. For the marker rs109163366 in *PPARGC1A* gene, allele A was higher in the three selection lines. Fisher's exact test revealed significant differences in the allele frequency of markers in *ADIPOQ* and *PPARGC1A* genes between the NeC and NeT lines. The allele frequencies of markers *ADIPOQ* and *OLR1* genes differed significantly between the NeS and NeT lines, whereas for marker in *PPARGC1A* gene a significant difference was only observed between the NeC and NeS lines (Table 3).

The substitution allelic effect of each SNP and its standard error, the P-values and the percentage of the additive genetic variance explained were estimated for each studied trait (Table 4).

A significant effect (P < 0.05) between the rs109019599 marker in *OLR1* gene and the traits was observed for W210 and RF (Table 4). This is the first result of a significant association between a marker in *OLR1* gene with carcass traits in cattle. Other significant associations were just observed in milk production traits (Ates et al., 2014, Wang et al., 2012, Wang et al., 2013). The finding of our study provides support that genes acting on basal fat metabolism are potential molecular markers both for milk fat content and for carcass fat deposition. This SNP is responsible for 1.79% and 1.06% of the additive genetic variance of the traits W210 and RF, respectively. It means that the inclusion of these SNPs in a low density SNP chip could contribute to increase the accuracy of genetic merit predictions in Nelore cattle. The OLR1 gene acts in low-density lipoprotein degradation as well as in glucose and lipid metabolism in liver (Khatib et al., 2006), it is clear to verify its influence in RF, a trait that is related to fat deposition. It is interesting to analyze that it is also important for weaning weight (W210), suggesting that the lipid metabolism might be important for the trait.

Significant effects were also observed between the rs109163366 in *PPARGC1A* gene and W378 (P < 0.05) explaining 1.12% of the additive genetic variance of the trait. Polymorphisms in the same gene were also correlated with growth and carcass traits in other beef cattle breeds (Li et al., 2014, Sevane et al., 2013, Shin and Chung, 2013). The absence of association with carcass traits was also observed by Tizioto et al. (2012) working with the same breed. The *PPARGC1A* is a metabolic switch that influences the mitochondrial, lipid and glucose metabolism, the significant association is an indication that the fat metabolism participates in weight at 378 days in female cattle.

No association was observed for the polymorphism located in the *ADIPOQ* gene. Although, the gene means to influence similar traits in other cattle breeds (Barendse, 2011, Shin and Chung, 2013, Zhang et al., 2013, Zhang et al., 2014); the absence of association might be explained by the genetic constitution of the breeds. How the genetic constitution is different, the effect of the alleles and their interactions may change as well as the contribution of them in the additive variance.

## Conclusion

In conclusion, the polymorphisms in the *ADIPOQ*, *OLR1* and *PPARGC1A* genes previously described for *Bos taurus taurus* were also identified in *B. taurus indicus*. The significant markers are very good candidates to be incorporated in assisted selection. Studies investigating other regions of these genes, the validation of these markers in another population of the same breed and their influence in other production traits are interesting to be done in the future.

## Figures and Tables

**Table 1:** Mean of each trait obtained for the three lines.

Trait	NeC		NeS		NeT	
Mean	SD	Mean	SD	Mean	SD
BW (kg)	25.12	(3.6)	32.05	(5.3)	32.23	(4.9)
W210 (kg)	158.54	(24)	190.7	(30.7)	197.80	(30.1)
W550 (kg)	253.86	(23.8)	312.55	(33.1)	315.03	(42.3)
H550 (cm)	127.42	(3.9)	134.81	(3.9)	134.15	(4.6)
H378 (cm)	125.05	(2.8)	133.16	(5.0)	133.01	(4.1)
W378 (kg)	280.15	(25.2)	351.84	(36.3)	357.12	(37.2)
LEA (cm^2^)	45.41	(5.5)	46.64	(7.3)	49.36	(6.9)
BF (mm)	1.67	(0.6)	1.76	(0.6)	1.78	(0.8)
RF (mm)	3.98	(1.9)	4.39	(1.9)	4.51	(1.9)

**Table 2:** Markers allele and genotype frequencies in the three Nelore lines selected for growth.

Marker (gene)/line	Genotype frequency			Allele frequency	
rs208549452 (ADIPOQ)	TT	TC	CC	T	C
NeC	0.41	0.24	0.35	0.53	0.47
NeS	0.42	0.22	0.36	0.53	0.47
NeT	0.34	0.16	0.50	0.42	0.58
rs109019599 (*OLR1*)	AA	AC	CC	A	C
NeC	0.08	0.20	0.72	0.17	0.83
NeS	0.03	0.14	0.82	0.11	0.89
NeT	0.12	0.17	0.70	0.21	0.79
rs109163366 (*PPARGC1A*)	AA	AC	CC	A	C
NeC	0.69	0.17	0.13	0.78	0.22
NeS	0.49	0.38	0.13	0.68	0.32
NeT	0.39	0.49	0.12	0.63	0.37

**Table 3:** Comparison of allelic frequencies between the selection lines (P value, Fisher's exact test).

	P-value		
Population pair	rs208549452 (*ADIPOQ*)	rs109019599 (*OLR1*)	rs109163366 (*PPARGC1A*)
NeC vs NeT	< 0.007	0.42	< 0.0003
NeC vs NeS	0.92	0.27	< 0.01
NeS vs NeT	< 0.0002	< 0.007	0.19

**Table 4:** SNP association analysis in cattle population.

Traits	Markers	P	Named allele	Effect	SE	%Va
BW (kg)	*ADIPOQ/Bsr*I	0.08	C	− 0.57	0.20	0.32
W210 (kg)	*ADIPOQ/Bsr*I	0.91	C	0.08	0.06	0.00
W550 (kg)	*ADIPOQ/Bsr*I	0.77	C	− 0.19	0.07	0.01
H550 (cm)	*ADIPOQ/Bsr*I	0.23	C	− 0.46	0.14	0.10
W378 (kg)	*ADIPOQ/Bsr*I	0.13	C	− 0.63	0.12	0.20
H378 (cm)	*ADIPOQ/Bsr*I	0.25	C	− 0.38	0.09	0.39
LEA (cm^2^)	*ADIPOQ/Bsr*I	0.65	C	− 0.10	0.07	0.03
RF (mm)	*ADIPOQ/Bsr*I	0.57	C	0.03	0.02	0.06
BF (mm)	*ADIPOQ/Bsr*I	0.38	C	0.10	0.05	0.10
BW (kg)	*OLR1/Pst*I	0.15	A	− 0.19	0.11	0.19
**W210 (kg)**	***OLR1/Pst*I**	**0.02**	**A**	**− 2.57**	**1.51**	**1.79**
W550 (kg)	*OLR1/Pst*I	0.13	A	− 2.86	2.05	0.21
H550 (cm)	*OLR1/Pst*I	0.39	A	− 0.30	0.18	0.13
W378 (kg)	*OLR1/Pst*I	0.10	A	− 0.26	0.09	0.24
H378 (cm)	*OLR1/Pst*I	0.26	A	− 0.31	0.13	0.18
LEA (cm^2^)	*OLR1/Pst*I	0.14	A	− 0.74	0.38	0.32
**RF (mm)**	***OLR1/Pst*I**	**0.04**	**A**	**− 0.09**	**0.02**	**1.06**
BF (mm)	*OLR1/Pst*I	0.88	A	0.02	0.01	0.00
BW (kg)	*PPARGC1/Nhe*I	0.11	A	− 0.34	0.17	0.24
W210 (kg)	*PPARGC1/Nhe*I	0.15	A	− 2.03	1.13	0.95
W550 (kg)	*PPARGC1/Nhe*I	0.79	A	− 0.07	0.04	0.00
H550 (cm)	*PPARGC1/Nhe*I	0.70	A	− 0.16	0.07	0.01
**W378 (kg)**	***PPARGC1/Nhe*I**	**0.02**	**A**	**− 0.98**	**0.11**	**1.12**
H378 (cm)	*PPARGC1/Nhe*I	0.14	A	− 0.59	0.09	0.64
LEA (cm^2^)	*PPARGC1/Nhe*I	0.56	A	− 0.14	0.06	0.09
RF (mm)	*PPARGC1/Nhe*I	0.53	A	0.04	0.01	0.07
BF (mm)	*PPARGC1/Nhe*I	0.53	A	0.03	0.03	0.02

The bold emphasis means the significant markers (p < 0.05).

Significance (P), allelic substitution effect (effect) for the named allele of each SNP, its standard error (SE) and the percentage of additive genetic variance (%Va) explained by the genotypes of each SNP on birth weight (BW), weaning weight adjusted to 210 days of age (W210), females yearling weight adjusted to 550 days of age (W550), female yearling hip height (H550), males yearling weight adjusted to 378 days of age (W378), male yearling hip height (H378), loin eye area (LEA), backfat thickness (BF) and rump fat thickness (RF).
